# Mitochondrial Dynamics in Diabetic Kidney Disease: Underlying Mechanisms and Novel Therapeutics

**DOI:** 10.3390/ijms27052429

**Published:** 2026-03-06

**Authors:** Nan Shao, Jinghan Wang, Jiaoying Liu, Junhua Zhang, Bin Zhang, Xiaobo Sun, Xiaoqiu Liu

**Affiliations:** 1 Pharmacology and Toxicology Center, Shenyang Pharmaceutical University, Shenyang 110016, China; 19104354417@163.com (N.S.); wjh233035@163.com (J.W.); liujiaoying2024@163.com (J.L.); 2 Institute of Medicinal Plant Development, Peking Union Medical College and Chinese Academy of Medical Sciences, Beijing 100193, China; 3 Institute of Traditional Chinese Medicine, Tianjin University of Traditional Chinese Medicine, Tianjin 301617, China; zjhtcm@foxmail.com; 4 Diabetes Research Center, Chinese Academy of Medical Sciences, Beijing 100193, China; 5 Key Laboratory of Efficacy Evaluation of Chinese Medicine Against Glycolipid Metabolism Disorder Disease, State Administration of Traditional Chinese Medicine, Beijing 100193, China; 6 Key Laboratory of Bioactive Substances and Resources Utilization of Chinese Herbal Medicine, Ministry of Education, Beijing 100193, China

**Keywords:** mitochondrial dynamics, diabetic kidney disease, mitophagy

## Abstract

Diabetic kidney disease (DKD) is a prevalent and serious complication of diabetes and a leading cause of end-stage renal disease (ESRD). As the central organelles for cellular energy metabolism, mitochondria play a pivotal role in the pathogenesis of DKD. Structural and functional impairments of mitochondria trigger multiple renal pathological processes, such as oxidative stress, apoptosis, chronic inflammation, and fibrosis. Mitochondrial dynamics are crucial for maintaining mitochondrial integrity, and their involvement in the progression of DKD is increasingly recognized. Nevertheless, comprehensive reviews addressing the relationship between mitochondrial dynamic homeostasis and DKD are still lacking. This review systematically summarizes the pivotal role of imbalanced mitochondrial dynamics in the pathogenesis and progression of DKD. It details the underlying regulatory mechanisms and stage-specific pathological contributions across different renal cell types, discusses potential diagnostic and therapeutic applications, and evaluates the prospects of natural products that target mitochondrial dynamics in DKD. By integrating current evidence, this work aims to provide a theoretical foundation and strategic guidance for innovative drug development and precision medicine in DKD.

## 1. Introduction

In recent years, global lifestyle and dietary change have driven a continuous increase in diabetes incidence, making it a major public health challenge worldwide [1]. The number of individuals with diabetes mellitus (DM) is projected to reach 537 million by 2021 and approximately 642 million by 2040. As a common microvascular complication, diabetic kidney disease (DKD), affects up to 21.8% of diabetic patients [2] and is a leading cause of end-stage renal disease (ESRD). Current prevention and management strategies for DKD primarily rely on glycemic and blood pressure control, yet they show limited efficacy in halting disease progression [3]. Due to the lack of effective treatments, most DKD patients eventually progress to ESRD, with a five-year survival rate generally below 20% [4]. Although advances have been made in both clinical and basic research on DKD, its pathogenesis remains incompletely understood, and targeted therapies are still lacking. Thus, further investigation into the molecular mechanisms underlying DKD and their renal implications is urgently needed. Existing evidence indicates that DKD involves multiple pathological processes, including dysregulated glucose metabolism, tubulointerstitial fibrosis, oxidative stress, and inflammation. Notably, mitochondrial dysfunction triggered by dysregulated glucose and lipid metabolism has recently emerged as a critical contributor to DKD pathogenesis.

Mitochondria are highly dynamic organelles that play a central role in cellular energy production and metabolism [5]. The kidney, as an energy-intensive organ, is particularly rich in mitochondria, and its functional integrity is closely related to mitochondrial homeostasis. This dependence is especially pronounced in proximal tubule cells, which rely predominantly on mitochondrial oxidative phosphorylation to generate ATP and sustain normal physiological activities [6]. The critical role of mitochondria in renal function is further evidenced by the renal impairment observed in patients with hereditary mitochondrial diseases [7].

In DKD, a hyperglycemic microenvironment has been shown to reduce mitochondrial membrane potential within renal endothelial cells and podocytes [8]. Under metabolic stresses such as hyperglycemia, mitochondrial overproduction of reactive oxygen species (ROS) activates multiple pathological pathways, including inflammation and apoptosis [9,10]. Furthermore, accumulating studies indicate that diabetes-related factors, particularly hyperglycemia, can induce significant alterations in mitochondrial morphology characterized by excessive fission and increased volume, which play a pivotal role in the pathogenesis of DKD. Studies using various experimental models of DKD have confirmed that high glucose-induced mitochondrial fission drives excessive ROS generation and cellular apoptosis [11]. Emerging evidence further suggests that dysregulated regulation of mitochondrial fusion may also play a role in the progression of DKD [12,13].

Accordingly, imbalance in mitochondrial dynamics has become a focus in DKD research, particularly regarding its specific mechanisms in renal injury. However, systematic summaries of mitochondrial dynamics imbalance in the progression of DKD remain scarce. Existing reviews tend to focus on isolated aspects of mitochondrial dynamics, often lacking a comprehensive integration of its role across different pathological stages and multiple renal cell types. To address this gap, this review systematically outlines the abnormal manifestations of mitochondrial dynamics in DKD, explores their specific impact on renal pathological structures, evaluates the potential of mitochondrial dynamics as a therapeutic target in DKD, and highlights the prospects of natural products that function through modulation of mitochondrial dynamics. By synthesizing current evidence, this work aims to provide a more comprehensive and targeted theoretical basis and strategic guidance for the diagnosis and treatment of DKD.

## 2. Mitochondrial Dynamics

Mitochondrial dynamics encompass the processes governing mitochondrial morphology, number, distribution, and function, primarily through mitochondrial fusion, fission, transport, biogenesis, and organelle interactions. These mechanisms are essential for maintaining cellular energy metabolism, stress adaptation, and survival [14]. As highly dynamic organelles, mitochondria vary widely in number and morphology across cell types; in fibroblasts, for instance, they range from dozens to hundreds in number and display diverse forms, including vesicles, short or elongated tubules, and interconnected networks. These structural states are dynamically regulated by the opposing processes of mitochondrial fusion and fission: while fusion promotes the formation of elongated and interconnected tubules, fission leads to mitochondrial fragmentation into smaller, discrete units [15]. Another crucial aspect of mitochondrial dynamics is mitophagy, which selectively removes damaged mitochondria to preserve organelle quality. Beyond energy production, mitochondria also critically regulate cell death, stress signaling, and metabolic homeostasis [16].

### 2.1. Mitochondrial Fission and Fusion

**Figure 1:**
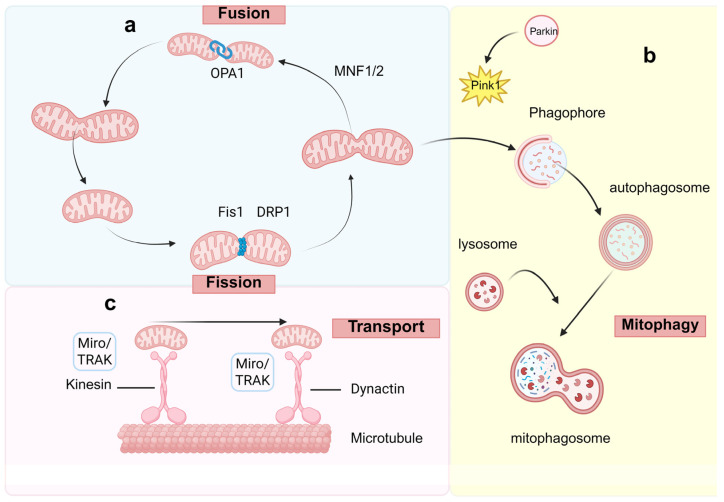
Mitochondrial homeostasis Mitochondria maintain cellular health through three key dynamic processes: fission/fusion, quality control (mitophagy), and intracellular trafficking. Dysregulation of these processes is central to the pathogenesis of diabetic kidney disease. (**a**) Mitochondrial fission and fusion. Mitochondria maintain a dynamic network through constant fission and fusion. Fission is primarily regulated by *Drp1*, while fusion is mediated by the outer membrane proteins *Mfn1*/*Mfn2* and the inner membrane protein *Opa1*. The balance between these processes is crucial for cellular homeostasis. (**b**) Clearance of damaged mitochondria. Upon mitochondrial damage, *PINK1* accumulates on the outer membrane and activates *Parkin*. *Parkin* ubiquitinates damaged mitochondria, marking them for recognition by autophagic receptors. This leads to engulfment by autophagosomes and subsequent degradation via lysosomes. (**c**) Mitochondrial trafficking. Mitochondria are transported along microtubules via the TRAK-Miro adaptor complex, which links mitochondria to molecular motors such as kinesin, enabling directed movement to meet localized cellular energy demands. Created in BioRender. Zhang, B. (2026) https://BioRender.com/s0tiw4z (accessed on 1 December 2025).

Mitochondrial fusion refers to the process whereby two or more mitochondria merge their membranes to form a larger, interconnected structure. This process facilitates the functional recovery of damaged mitochondria and helps maintain the stability of mitochondrial DNA (mtDNA). By enabling content exchange between mitochondria, fusion serves to balance the energy states across the mitochondrial network, thereby enhancing its overall functional capacity [17]. In contrast, mitochondrial fission involves the division of a single mitochondrion into two or more smaller units. This process allows cells to dynamically regulate mitochondrial number, distribution, and morphology, and plays a key role in optimizing mitochondrial function under varying physiological demands (Figure 1a). For instance, under conditions of high energy demand, fission can increase mitochondrial numbers to meet cellular requirements [18]. Together, mitochondrial fission and fusion are essential for maintaining mitochondrial morphology and functional integrity. While fission facilitates the removal of damaged mitochondria through segregation and subsequent mitophagy, fusion supports functional complementation and enhances cellular resilience to stress [19]. In diabetes, both fission and fusion processes are frequently impaired, leading to mitochondrial dysfunction that contributes to the progression of renal injury [20].

### 2.2. Mitochondrial Transport and Localization

The intracellular distribution of mitochondria is a tightly regulated process that depends primarily on the microtubule cytoskeleton. Directed movement is achieved through molecular motors (kinesin, dynein) and adaptor complexes such as Miro/TRAK, which ensure that mitochondria are delivered to subcellular sites with elevated energy requirements or localized stress [21] (Figure 1c).

In renal tissue, this spatial control carries particular physiological relevance. tubular epithelial cells exhibit a high dependence on mitochondrial oxidative phosphorylation (OXPHOS) to produce the ATP needed for active solute reabsorption. Accordingly, mitochondria are enriched at the basolateral membrane, where they are positioned to support ion-pump activity and maintain tubular function [22]. Likewise, in glomerular podocytes, proper cytoskeletal organization and slit-diaphragm integrity rely on locally available ATP, underscoring the importance of correct mitochondrial positioning [23].

Under diabetic conditions, mitochondrial trafficking is often compromised. Persistent hyperglycemia, advanced glycation end-product (AGEs) accumulation, and oxidative stress can destabilize microtubules, impair motor-protein function, and disrupt adaptor-protein modifications. As highlighted by Higgins and Coughlan, mitochondrial dysfunction lies at the heart of DKD pathogenesis, and defective mitochondrial transport likely amplifies this pathology. Impaired trafficking limits the supply of functional mitochondria to energy-demanding domains (e.g., the basolateral membrane of tubules) and hampers the removal of damaged organelles to autophagic sites. The consequent local ATP shortfall, together with the accumulation of dysfunctional mitochondria, establishes a self-reinforcing cycle of bioenergetic failure and oxidative injury. Thus, dysregulation of mitochondrial transport and localization is not merely a manifestation of mitochondrial network disruption in DKD, but may actively contribute to tubular dysfunction, defective protein handling, and deterioration of the glomerular filtration barrier [22].

### 2.3. Mitophagy

Mitophagy is a specialized form of cellular autophagy, referring to the process by which cells eliminate damaged or redundant mitochondria through autophagic mechanisms. This process serves as a crucial mechanism for maintaining cellular homeostasis, removing damage, and preserving energy metabolism balance [24,25]. Autophagy is fundamentally a degradation process, involving the encapsulation of cellular waste or damaged organelles within a double-membrane structure (autophagosome), followed by degradation via lysosomes or vacuoles (Figure 1b). Mitophagy plays a critical role in maintaining mitochondrial health and eliminating damaged mitochondria [26,27]. Research indicates that impaired mitophagy is closely associated with fibrosis development. Damaged mitochondria accelerate renal damage progression by releasing increased inflammatory mediators and promoting fibrotic responses [28]. Activating mitophagy to clear damaged mitochondria can alleviate the fibrotic process in DKD [29].

## 3. Abnormal Manifestations of Mitochondrial Dynamics in Diabetic Kidney Disease

### 3.1. Impaired Mitochondrial Fusion

In renal tissues from patients with diabetic kidney disease (DKD) and experimental animal models, the expression of key mitochondrial fusion proteins, including Mitofusin 2 (*Mfn2*) and Optic Atrophy 1 (*Opa1*), is frequently observed to be downregulated [30,31]. This molecular change appears to correlate with structural abnormalities in the mitochondrial network. Transmission electron microscopy (TEM) analyses in podocyte-specific *Mfn2* knockout or inhibition models, for example, have shown increased mitochondrial fragmentation, disrupted network connectivity, and impaired mitochondria-endoplasmic reticulum contact sites (MAMs), suggesting that *Mfn2* may contribute to the maintenance of mitochondrial morphology and MAM integrity [32]. Available evidence indicates that hyperglycemia-induced oxidative stress could be a central mechanism underlying these alterations [33], as it promotes the excessive production of reactive oxygen species (ROS) [34], which are known to damage cellular components and potentially exacerbate mitochondrial dysfunction [35]. Additionally, oxidative stress has been reported to activate signaling pathways such as NF-κB and MAPK, which may further suppress *Mfn2* and *Opa1* expression and thereby contribute to impaired mitochondrial fusion [36].

Studies using high-sugar-induced Drosophila models of DKD further suggest that inhibition of the *PINK1*-*Parkin* pathway may lead to reduced mitophagy and fusion capacity. Corresponding TEM observations in these models reveal disrupted mitochondrial networks, increased fragmentation, and ultrastructural alterations—including cristae disorganization and mitochondrial swelling—which visually reflect possible disturbances in mitochondrial dynamics under hyperglycemic conditions [37]. Therefore, mitochondrial fusion is generally regarded as important for maintaining functional integrity, supporting energy metabolism, and facilitating cellular stress adaptation [38]. When *Mfn2* and *Opa1* expression is decreased, the fusion process may be compromised, leading to excessive mitochondrial fission and fragmentation. These changes are thought to reduce energy production efficiency and disrupt metabolic homeostasis, thereby potentially contributing to the progression of DKD [39].

### 3.2. Abnormal Increase in Mitochondrial Fission

During the progression of diabetic kidney disease (DKD), dynamin-related protein 1 (*Drp1*), a key regulator of mitochondrial fission, has been frequently observed to show elevated expression and activity, which may contribute to disease progression [40,41]. Ultrastructural evidence from transmission electron microscopy (TEM) appears to support this observation, revealing increased mitochondrial fragmentation—characterized by a greater prevalence of short, spherical mitochondrial profiles compared to the typical tubular network—in renal biopsy samples from DKD patients and in podocytes of diabetic animal models [42,43]. On a mechanistic level, hyperglycemia is thought to facilitate the translocation of *Drp1* from the cytosol to the mitochondrial outer membrane via several signaling pathways, potentially enhancing its fission activity [44]. In vivo investigations further suggest that podocyte-specific *Drp1* knockout can improve mitochondrial morphology in the glomeruli of diabetic mice. TEM analyses in these models indicate a reduction in mitochondrial fragmentation and a tendency toward more elongated mitochondrial forms, changes that correlate with improvements in renal function and reductions in proteinuria [45]. Consequently, the overactivation of *Drp1* is considered to promote excessive mitochondrial fission, which may compromise both structural and functional integrity [46]. Beyond inducing morphological alterations, excessive mitochondrial fission has been linked to impairments in energy metabolism and respiratory chain function. The disintegration of the mitochondrial network into smaller, less efficient units appears to diminish oxidative phosphorylation capacity, reduce ATP synthesis, and increase metabolic stress in renal cells, notably podocytes and tubular epithelial cells [47,48].

### 3.3. Impaired Mitophagy in DKD

In diabetic kidney disease (DKD), mitophagy is often functionally impaired. Persistent hyperglycemia and associated metabolic disturbances subject mitochondria to elevated oxidative stress and increased generation of reactive oxygen species (ROS). These reactive molecules can progressively damage mitochondrial membranes and components, contributing to a decline in mitochondrial function [49,50]. Under physiological conditions, such damage typically triggers efficient mitophagic clearance; however, in DKD, this quality-control mechanism appears compromised. Consequently, dysfunctional mitochondria may accumulate, perpetuating oxidative injury and establishing a potential vicious cycle that exacerbates renal cellular damage [24,51].

At the molecular level, DKD is associated with dysregulation in the expression and activity of key autophagy-related proteins. The mitophagy process depends on the coordinated action of proteins such as Atg family members, *LC*3, *PINK1*, and *Parkin*, which mediate the recognition, labeling, and degradation of damaged mitochondria [52,53,54]. Among these, the “*PINK1*/*Parkin* pathway” is regarded as a central regulator of mitophagy. In vitro studies using podocytes and tubular epithelial cells indicate that high-glucose conditions can downregulate the expression of *PINK1* and *Parkin*, reduce the stability of *PINK1* on the mitochondrial membrane, and impair *Parkin* recruitment, thereby attenuating the initiation of mitophagy. Supporting these observations, animal models of diabetes show that reduced activity of the *PINK1*/*Parkin* pathway correlates with diminished mitophagic flux and aggravated renal injury. Conversely, pharmacological activation of this pathway has been reported to enhance mitophagy and alleviate pathological changes in the diabetic kidney, suggesting that *PINK1*/*Parkin* signaling may play an important role in DKD progression. Moreover, chronic hyperglycemia can disrupt upstream regulatory circuits of autophagy. In vitro evidence indicates that the mTOR pathway is frequently hyperactivated under diabetic conditions, which may suppress autophagic flux and compromise the clearance of damaged mitochondria. Consistent with this, animal studies further demonstrate that sustained mTOR activation not only inhibits mitophagy but may also weaken intrinsic repair mechanisms, rendering renal tissue more vulnerable to oxidative and inflammatory injury, thereby contributing to the progression of DKD.

### 3.4. Morphological Changes in Mitochondria

Altered mitochondrial morphology is commonly observed in DKD [52]. Under physiological conditions, mitochondria typically exhibit a well-organized ultrastructure, featuring a smooth outer membrane and a highly folded inner membrane that forms cristae—a morphology crucial for efficient energy production [55]. In the context of DKD, however, sustained hyperglycemia and associated oxidative stress are thought to promote structural disorganization, which may include cristae loss, membrane disruption, mitochondrial swelling, and fragmentation. Such morphological changes are often correlated with functional deficits, as misshapen mitochondria tend to show reduced energy-transduction capacity, thereby contributing to disturbances in cellular bioenergetics [56]. In addition to driving structural alterations, persistent hyperglycemia may further exacerbate mitochondrial dysfunction [57]. The accumulation of mitochondrial damage can disrupt intracellular calcium homeostasis, interfere with glucose and lipid metabolism, and has been linked to the activation of apoptotic pathways [58,59]. In key renal cell types affected in DKD, including tubular epithelial cells and glomerular cells, mitochondrial injury is considered a central contributor to functional decline, inflammatory activation, and the progression of renal fibrosis [60].

**Figure 2:**
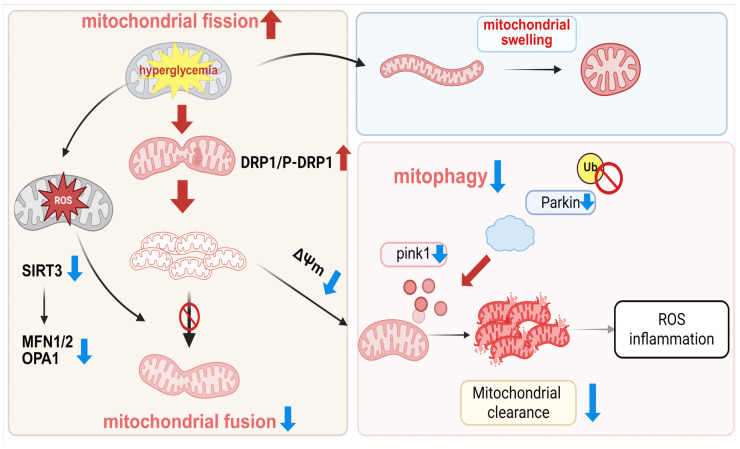
Hyperglycemia disrupts mitochondrial dynamics and quality control in DKD. Sustained hyperglycemia promotes mitochondrial fission (↑) through upregulation of *Drp1* and phospho-*Drp1* while suppressing mitochondrial fusion (↓) via downregulation of *Mfn1*, *Mfn2*, and *Opa1*. These imbalances lead to mitochondrial network fragmentation. Concurrently, impaired *PINK1*/*Parkin*-mediated mitophagy (↓) and reduced ubiquitination (Ub ↓) prevent the clearance of damaged mitochondria. The resulting accumulation of dysfunctional mitochondria induces morphological abnormalities (e.g., swelling), elevated ROS production (↑), and exacerbation of oxidative stress and inflammatory responses, collectively driving renal injury. Created in BioRender. Zhang, B. (2026) https://BioRender.com/yzzyhro (accessed on 3 December 2025).

In summary, hyperglycemia disrupts mitochondrial homeostasis by impairing the balance between fusion and fission, leading to mitochondrial swelling, functional deficits, and compromised autophagic clearance. These alterations ultimately elevate reactive oxygen species (ROS) production and promote inflammatory responses. Together, these interconnected processes delineate the aberrant mitochondrial dynamics observed in DKD (Figure 2). However, while we seek to understand these phenomena and devise targeted strategies, we must also recognize their inherent complexity and context-dependent nature.

First, while substantial evidence supports the nephroprotective effects of inhibiting excessive fission or promoting fusion, simplistically labeling mitochondrial fission as “harmful” may be misleading. Studies indicate that *Drp1*-mediated moderate fission is a crucial step required to initiate specific types of mitophagy, including pathways partially independent of *Parkin*. Studies in neurons and cardiomyocytes reveal that complete inhibition of *Drp1* actually impairs mitochondrial quality control, leading to the accumulation of dysfunctional organelles [53]. This suggests that therapeutic goals should focus on restoring the physiological equilibrium and precise regulation of the dynamic network, rather than pursuing absolute inhibition or promotion of any single process.

Second, conclusions regarding the specific roles of key regulatory proteins like *Drp1*, *Opa1*, and *Mfn2* in DKD are not always consistent across different research systems. This inconsistency may stem from variations in disease stage, cell type, experimental model (e.g., in vivo/in vitro), and detection methods employed. More intriguingly, recent studies have even demonstrated in tumor cells that forcibly promoting fusion (e.g., by overexpressing *Mfn2*) may lead to abnormally enhanced mitophagy, impaired OXPHOS function, and subsequent damage to cellular energy metabolism [61]. This “contradictory” phenomenon profoundly reveals that the biological significance of mitochondrial fusion may be highly “context-dependent.” Its ultimate effect in specific pathological environments depends on the overall impact on mitophagy flux, biogenesis, and energy metabolism networks.

Therefore, future research urgently needs to move beyond observing single protein expression or morphological changes toward deeper, systematic exploration of underlying mechanisms. It is essential to precisely decipher the dynamic coupling between mitochondrial fission, fusion, and autophagy across different stages of DKD and diverse renal cell types. Developing more cell-specific intervention tools will validate the therapeutic concept of “restoring equilibrium” rather than “unidirectional intervention.” Only through such approaches can we fully comprehend the landscape of mitochondrial dynamics in DKD and translate this understanding into safe and effective clinical treatment strategies.

## 4. Key Roles of Abnormal Mitochondrial Dynamics in the Pathogenesis of Diabetic Kidney Disease

### 4.1. Mitochondrial Dynamics Abnormalities and Glomerular Injury

**Figure 3:**
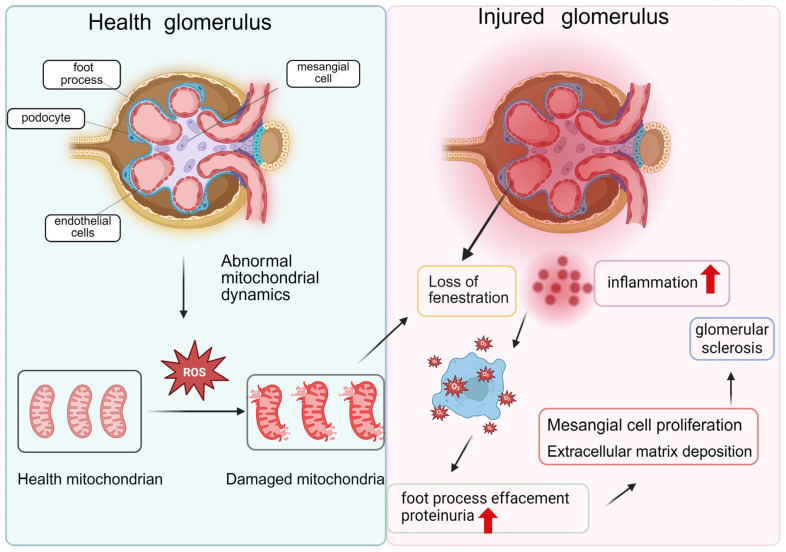
Comparative schematic of mitochondrial dynamics in glomerular health and injury. The left panel depicts a healthy glomerulus, wherein endothelial cells, mesangial cells, and podocytes maintain structural and functional integrity supported by a dynamic mitochondrial network. The right panel illustrates the consequences of abnormal mitochondrial dynamics under diabetic conditions. Mitochondrial fragmentation and dysfunction initiate a cascade of cellular injury: endothelial cells lose their characteristic fenestrations and exhibit pro-inflammatory activation; podocytes display effacement of foot processes, leading to loss of filtration selectivity and proteinuria; and mesangial cells undergo proliferation and excessive extracellular matrix deposition; These pathological changes collectively contribute to the progression of glomerulosclerosis. Created in BioRender. Zhang, B. (2026) https://BioRender.com/53rraod (accessed on 4 December 2025).

Studies have shown that renal cells, including podocytes, mesangial cells, and endothelial cells, exhibit significant dysregulation of mitochondrial dynamics during the progression of diabetic kidney disease (DKD). The following section discusses the relationship between mitochondrial dynamics abnormalities and glomerular injury from these three cellular perspectives (Figure 3).

#### 4.1.1. Podocyte Dysfunction

Diabetic kidney disease (DKD) is defined by three key pathological features: abnormal urinary albumin excretion, distinctive glomerular lesions, and a progressive decline in glomerular filtration rate [62,63]. Among these, podocyte injury is a central driver of DKD progression. Under diabetic conditions, podocytes sustain multi-level damage, beginning with a significant reduction in cell number during early disease stages, often preceding overt proteinuria [64]. The decline in podocyte numbers, reported as 20–40% in early DKD, compromises the glomerular filtration barrier. This loss is primarily driven by hyperglycemia-induced apoptosis, increased detachment from the glomerular basement membrane, and the limited regenerative capacity of podocytes [65,66].

Accumulating evidence highlights the crucial role of dysregulated mitochondrial dynamics in podocyte dysfunction. As terminally differentiated cells with high energy demands, podocytes rely on efficient oxidative phosphorylation, which requires precise control of mitochondrial dynamics [67,68]. In DKD, however, persistent hyperglycemia and dyslipidemia disrupt this balance, promoting excessive mitochondrial fission and suppressed fusion in podocytes [69]. Excessive fission leads to mitochondrial fragmentation, reducing ATP production and disrupting the architecture of foot processes. Impaired fusion, in turn, contributes to mitochondrial DNA instability, increased reactive oxygen species (ROS) generation, and activation of apoptosis, collectively accelerating podocyte loss and filtration barrier failure [43].

This disruption in mitochondrial dynamics initiates a pathological cascade: fragmentation compromises the electron transport chain, leading to substantial ROS accumulation and impaired ATP production. The energy deficit destabilizes the podocyte cytoskeleton, while sustained damage activates intrinsic apoptotic pathways, accelerating podocyte detachment from the glomerular basement membrane. Collectively, these alterations disrupt the structural and functional integrity of the glomerular filtration barrier, manifesting as progressive proteinuria and ultimately advancing toward irreversible glomerulosclerosis [43,70].

Notably, such abnormalities in mitochondrial dynamics often occur in early stages of DKD, preceding clinical symptoms [71]. These observations underscore the central role of mitochondrial dynamics in initiating podocyte injury and establish a critical framework for early intervention. Consequently, therapeutic approaches that correct the imbalance between mitochondrial fusion and fission or alleviate oxidative stress represent promising strategies to maintain podocyte health and delay the progression of DKD.

#### 4.1.2. Endothelial Dysfunction

Endothelial dysfunction is a pivotal factor in the progression of glomerulosclerosis and DKD [72]. As a critical component of the glomerular filtration barrier, highly specialized glomerular endothelial cells exhibit unique structural features essential for their function. Their surface is densely fenestrated, with pores significantly larger than albumin molecules and is coated by a complex luminal glycocalyx layer. This specialized structure not only endows endothelial cells with exceptional permeability and hydraulic conductivity to accommodate high glomerular filtration loads but also forms the primary selective barrier for macromolecules [73,74].

Nevertheless, the mechanisms of glomerular endothelial injury in diabetes are not fully understood. Notably, even in models of primary podocyte injury, endothelial cells display significant mitochondrial oxidative stress. Importantly, the endothelial mitochondrial dysfunction often precedes podocyte loss, suggesting it may be an early driver of glomerular damage [75]. Furthermore, endothelial dysfunction leads to albumin leakage, which exerts direct toxicity on podocytes. In diabetes, hyperglycemia disrupts the endothelial regulation of mitochondrial fission, which impairs the podocyte’s defense against this albumin toxicity. Concurrently, the altered microenvironment from the endothelial injury further promotes pathological mitochondrial fission in podocytes, aggravating the cellular damage [76].

More critically, hyperactivated mitochondrial fission initiates a self-sustaining pathological cycle. While mitochondrial fragmentation is often a consequence of initial oxidative stress, fragmented and dysfunctional mitochondria exhibit impaired electron transport chain function [77], which can further amplify reactive oxygen species (ROS) production through mechanisms such as ROS-induced ROS release (RIRR). RIRR is a self-amplifying mitochondrial oxidative stress mechanism: initial ROS (e.g., superoxide anion) induces mitochondrial membrane potential loss and opening of the mitochondrial permeability transition pore (mPTP), leading to the explosive release of mitochondrial ROS into the cytoplasm. These ROS further diffuse and activate the same process in adjacent mitochondria, forming a positive feedback loop that dramatically amplifies cellular oxidative damage [78]. These alterations not only directly damage endothelial cells but may also impair neighboring podocytes and mesangial cells (MCs) via paracrine signaling, collectively accelerating glomerulosclerosis [79]. In DKD, hyperglycemia activates fission-related proteins such as *Drp1*, disrupting the mitochondrial network and locking cells in a state of persistent oxidative stress that drives endothelial apoptosis and loss of glomerular filtration function [80]. These insights reveal new mechanistic underpinnings of the disease and highlight the therapeutic promise of targeting mitochondrial dynamics to preserve glomerular endothelial health and slow DKD progression.

#### 4.1.3. Mesangial Cell Dysfunction

Mesangial cells (MCs) are essential structural and functional components of the glomerulus, maintaining glomerular architecture and modulating filtration function. Through dynamic interactions with the glomerular basement membrane and endothelial cells, they form a supportive scaffold, and their contractile properties help regulate capillary hemodynamics and filtration rate [81]. Under physiological conditions, MCs tightly control extracellular matrix (ECM) homeostasis. In DKD, however, this balance is disrupted, leading to a dysfunctional state characterized by aberrant proliferation, a pro-inflammatory shift, and excessive ECM accumulation, which are key drivers of glomerulosclerosis.

Growing evidence highlights the central role of dysregulated mitochondrial dynamics in mesangial cell dysfunction. Under hyperglycemia, MCs exhibit a pronounced shift toward mitochondrial fission driven by increased *Drp1* and reduced fusion proteins (*Mfn2* and *Opa1*), resulting in network fragmentation. This structural disruption compromises the electron transport chain, leading to excessive ROS production. The ensuing oxidative stress damages mitochondrial integrity and activates inflammatory pathways including NF-κB and the NLRP3 inflammasome, fostering a pro-inflammatory glomerular microenvironment [82].

These changes establish a vicious cycle: fission-derived ROS promote abnormal MCs activation and proliferation, which in turn further aggravates mitochondrial impairment, accelerating the progression from mesangial expansion to sclerosis. Moreover, impaired mitophagy allows damaged mitochondria to accumulate, perpetuating ROS production and the release of pro-apoptotic factors. This ultimately increases MCs apoptosis and drives irreversible glomerular injury.

### 4.2. Tubular Injury and Mitochondrial Dynamics Dysregulation

**Figure 4:**
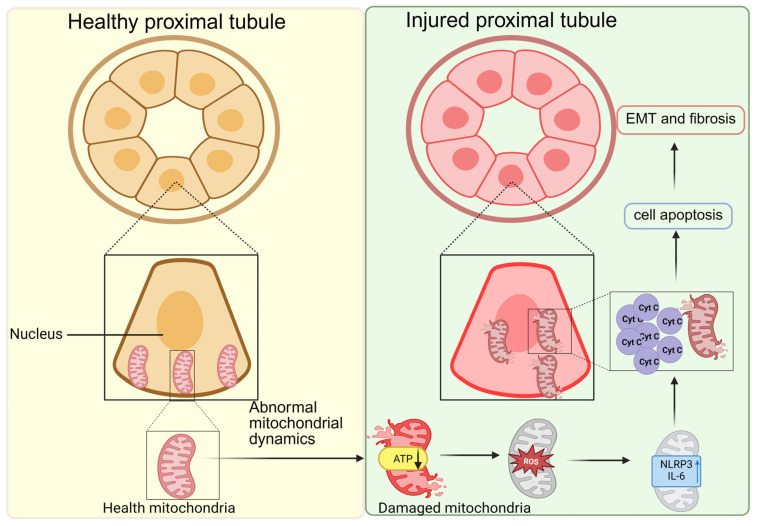
Hyperglycemia-induced mitochondrial dysfunction promotes tubular injury in diabetic kidney disease. Healthy tubular epithelial cells (**left**) exhibit structurally intact brush borders and tight junctions, with cytoplasm rich in interconnected mitochondrial networks. These structural features collectively support normal tubular reabsorption and secretory functions. Under prolonged hyperglycemia (**right**), abnormal mitochondrial dynamics occurs, characterized by fragmentation of the mitochondrial network. This leads to impaired ATP synthesis and increased ROS production. Elevated ROS further activates pro-inflammatory factors (e.g., IL-6) and inflammasomes (e.g., NLRP3), promoting inflammatory responses. Concurrently, increased cytochrome c release activates apoptotic pathways, ultimately inducing epithelial-mesenchymal transition (EMT) and renal interstitial fibrosis. Created in BioRender. Zhang, B. (2026) https://BioRender.com/2r3qw19 (accessed on 5 December 2025).

Tubular injury plays a clinically significant role in DKD, and is closely associated with albuminuria and impaired metabolic function [83] (Figure 4). Tubular damage often serves as a more accurate predictor of renal functional decline than glomerular alterations, highlighting its prognostic importance. Pathological studies further reveal widespread apoptosis in diabetic kidneys, with tubular epithelial cells showing notable susceptibility to the hyperglycemic milieu, a selective vulnerability that points to distinct underlying molecular mechanisms [84,85].

Rap1 has emerged as a key renoprotective mediator in DKD. Its expression is downregulated in tubular epithelial cells under diabetic conditions, Deficiency of Rap1 exacerbates high glucose-induced mitochondrial fragmentation, transforming the network from interconnected tubules into dispersed granules. These structural changes are accompanied by loss of mitochondrial membrane potential and cytochrome c release, collectively accelerating tubular apoptosis. Conversely, tubular-specific overexpression of Rap1 attenuates mitochondrial fragmentation, preserves mitochondrial morphology, reduces cytochrome c leakage and apoptosis, and ultimately ameliorates tubular injury in diabetic models, establishing Rap1 as a critical regulator of mitochondrial dynamics with significant therapeutic potential [86].

Disrupted mitochondrial dynamics drive pathological ROS overproduction under hyperglycemia. Impaired the electron transport chain leads to ROS accumulation, which causes direct cellular damage and initiates inflammatory responses by upregulating cytokines (e.g., TNF-α, IL-6) and activating the NLRP3 inflammasome activation, thereby promoting release of IL-1β and IL-18 and amplifying renal interstitial injury [87]. Elevated ROS and inflammatory mediators also activate multiple signaling pathways that upregulate key epithelial-mesenchymal transition (EMT) transcription factors, including Snail, Slug, and ZEB1. These factors bind directly to the E-cadherin promoter, repressing its transcription and sharply reducing the synthesis of intercellular junction proteins. Concurrently, they upregulate mesenchymal markers such as N-cadherin, vimentin, fibronectin, and α-smooth muscle actin (α-SMA), causing tubular epithelial cells to lose polarity and junctional integrity, and adopt a spindle-shaped mesenchymal morphology. The EMT of tubular epithelial cells leads to a loss of normal function, detachment, and interstitial migration, culminating in tubular atrophy and structural destruction. Alongside pathological matrix deposition, these changes constitute the core drivers of renal interstitial fibrosis, propelling DKD toward irreversible end-stage renal disease [88,89].

Overall, endothelial cell injury constitutes the initial trigger in the pathogenesis of DKD [90]. Hyperglycemia-induced damage to glomerular endothelial cells results in excessive mitochondrial ROS production, leading to glycocalyx shedding, upregulation of adhesion molecules, enhanced inflammatory infiltration, and disruption of endothelial-podocyte crosstalk [91,92]. Podocyte injury subsequently emerges as the central hub of glomerular damage. Increased vascular permeability due to endothelial dysfunction promotes the filtration of excess albumin and other toxic substances, which directly impair podocyte integrity [70]. Within podocytes, dysregulated mitochondrial dynamics—characterized by enhanced fission and suppressed fusion—compromises energy metabolism and intensifies oxidative stress, downregulating podocyte-specific proteins such as nephrin and podocin, inducing foot-process effacement, and ultimately triggering podocyte apoptosis or detachment [77]. This podocyte-derived injury, together with endothelial-derived inflammatory signals, further activates MCs. Mitochondrial dysfunction in MCs amplifies ROS generation and activates pathways including the NLRP3 inflammasome, driving a phenotypic switch from quiescence to activation [77]. Consequently, activated MCs undergo abnormal proliferation and overproduce extracellular matrix, resulting in progressive mesangial expansion—the structural hallmark of irreversible glomerulosclerosis [93]. The combined damage to the glomerular triad (endothelium-podocyte-mesangium) impairs filtration and induces proteinuria, while the filtered proteins and associated metabolic and ischemic stressors directly promote tubular epithelial injury and interstitial fibrosis, thereby driving the progression of DKD toward end-stage renal disease [94] (summarized in Table 1).

## 5. Therapeutic Agents Targeting Mitochondrial Dynamic Homeostasis in DKD

Given the pivotal role of mitochondrial dynamic imbalance in the progression of diabetic kidney disease (DKD), targeting this process has emerged as a promising therapeutic strategy. A growing number of agents capable of modulating mitochondrial fission, fusion, and mitophagy have shown potential in preclinical studies. Based on their chemical composition and origin, therapeutic agents targeting mitochondrial dynamics in DKD can be broadly classified into the following categories: structurally defined and mechanistically well-characterized active monomeric compounds; natural extracts with relatively complex compositions that act through multi-pathway synergy; traditional Chinese medicine formulas that emphasize holistic regulation and multi-target intervention; and rationally designed synthetic mitochondrial-targeted drugs developed based on specific molecular mechanisms (summarized in Table 2, Table 3, Table 4 and Table 5).

### 5.1. Monomeric Compounds

P110, a peptide inhibitor that specifically disrupts the *Drp1*-Fis1, interaction has shown therapeutic potential in a DKD animal model. Treatment with P110 reduces proteinuria, improves renal function, and alleviates histopathological injury. Mechanistically, P110 inhibits *Drp1* translocation to mitochondria and its binding to Fis1, thereby suppressing excessive mitochondrial fission. This effect helps maintain mitochondrial membrane potential and prevent Bax recruitment to mitochondria. Consequently, P110 blocks mitochondrial damage-induced double-stranded DNA release and inhibits activation of the cGAS-STING pathway, effectively attenuating the downstream inflammatory cascade [95].

High Glucose-Induced Protein-1 (IHG-1), a conserved mitochondrial protein upregulated in DKD, has been reported to play a crucial role in regulating mitochondrial dynamics and function. It enhances mitochondrial fusion and promotes mitochondrial biogenesis by amplifying the transforming growth factor-beta 1 (TGF-β1) signaling, thereby maintaining mitochondrial homeostasis. Studies show that inhibiting endogenous IHG-1 impairs mitochondrial respiration, reduces ATP production, suppresses mitochondrial fusion [96]. In contrast, IHG-1 overexpression promotes mitochondrial fusion, improves mitochondrial function, functional connections, protects against oxidative stress-induced apoptosis, and maintains cellular bioenergetics. Thus, IHG-1 is a vital regulator of mitochondrial health and cellular resilience in DKD.

Metformin, a first-line therapeutic drug for type 2 diabetes, exerts direct renoprotective effects in DKD beyond its glucose-lowering action. It reduces proteinuria and serum creatinine, and alleviates tubulointerstitial injury, oxidative stress and renal fibrosis in DKD model [97]. Mechanistically, metformin activates the AMPK pathway and upregulates autophagy proteins, thereby restoring impaired mitophagy in tubular epithelial cells under hyperglycemia. It promotes autophagic flux and reestablishes mitochondrial homeostasis, thereby ameliorating cellular dysfunction and delaying the progression of renal damage [98]. These integrated mechanisms provide a solid rationale for metformin’s beneficial effects on renal structure and function in DKD.

Inositol (MI), a key intracellular osmolyte and signaling molecule, participates in multiple signaling networks. Its metabolic disturbance in diabetes is closely associated with the progression of diabetic complications. In diabetic rat models, MI supplementation significantly reduces albuminuria and improved renal function. Mechanistically, MI restores impaired mitophagy by activating the *PINK1*/*Parkin* pathway and upregulating the autophagy receptor *PHB2* and *NIX*. MI also promotes mitochondrial biogenesis by activating the *Nrf2*/*SIRT1*/*PGC-1α* signaling [99]. Together, these synergistic actions elucidate MI’s renoprotective effects, supporting its therapeutic potential in DKD.

Melatonin, synthesized in both the pineal gland and peripheral tissues such as the kidney, exhibits renoprotective effects in DKD [100,101]. Mechanistically, melatonin promotes AMPK phosphorylation, which enhances the mitochondrial translocation of *PINK1* and *Parkin* to activate mitophagy [102]. This restored mitophagy reduces oxidative stress and inflammatory activation, thereby underpinning its therapeutic potential.

Asiatic acid, a pentacyclic triterpenoid from Centella asiatica alleviates tubular epithelial cells injury in DKD models by restoring mitochondrial dynamic homeostasis. It acts as a potent transcription factor Nrf2 activator, thereby downregulating the fission protein *Drp1* and upregulating the fusion protein *Mfn2*. Treatment with Asiatic acid rebalances mitochondrial dynamics, counteracts hyperglycemia-induced fragmentation, and improves bioenergetic function. Consequently, apoptotic signaling is attenuated, and tubular injury progression is ameliorated after the drug intervention [103].

**Table 2:** Monomeric Compounds Targeting Mitochondrial Dynamic Homeostasis in Diabetic Kidney Disease.

Drug-	Model	Mechanism	Influence	References
P110	Bilateral renal artery ligation in *C57BL/6J* male mice	*PGC-1α* ↑ *SIRT3* ↑ *Drp1* ↓ *Bax* ↓	Decreases the expression of inflammatory factors, inhibits excessive mitochondrial fission, and reduces the translocation of Bax to mitochondria	[95]
	ATP-depleted HK-2 cells LPS-treated HK-2 cells	*Drp1* ↓ *Bax* ↓	Reduces cell apoptosis, restores mitochondrial function, and decreases the expression of inflammatory factors.	
IHG-1	HG-induced HK-2 cells	*Mfns* ↑	Reduce apoptosis, promote mitochondrial fusion	[96]
Metformin	HFD+STZ-induced male *C57BL/6J* mice	*PINK1* ↑ *parkin* ↑	Lower blood sugar, reduce proteinuria, reduce oxidative stress, and improve kidney damage.	[98]
	HG-induced HK-2 cells	*Atg5* ↑ *LC3II* ↑	Reduce cell apoptosis, promote mitophagy	[99]
MI	STZ-induced male *SD* rats	*PINK1* ↑ *Parkin* ↑ *PHB2* ↑ *NIX* ↑ *Nrf2* ↑ *SIRT1* ↑ *PGC-1α* ↑	Alleviates renal oxidative stress inflammatory response, promotes mitochondrial biogenesis, improves mitochondrial functional homeostasis.	
	HG-induced NRK 52E cells	*ATP* ↑ *PINK1* ↑ *Parkin* ↑ *PHB2* ↑, *NIX* ↑ *Nrf2* ↑ *SIRT1* ↑ *PGC-1α* ↑	Activates mitophagy, increases ATP production, improves mitochondrial respiratory function, reduces mitochondrial fragmentation.	
Melatonin	HFD+STZ-induced male *C57BL/6J* mice	*PINK1* ↑ *parkin* ↑ *Lc3II* ↑ *P62* ↓ *p-AMPK* ↑	Improves kidney damage, inhibits inflammatory factors, alleviates kidney fibrosis, and activates mitophagy.	[102]
	HG-induced HK-2 cells	*Mfn-2* ↑ *PINK1* ↑ *parkin* ↑ *Lc3II* ↑ *P62* ↓ *Drp1* ↓	Inhibits inflammatory factors and improves cellular oxidative stress	
Asiatic Acid	STZ-induced male *SD* rats	*Drp1* ↓ *Mfn1* ↑ *Nrf-2* ↑ *Mfn2* ↑ *Keap-1* ↓ *HO-1* ↑	Lowers blood glucose, improves renal function, and regulates mitochondrial homeostasis.	[103]
	AGEs-induced HK-2 cells	*Drp1* ↓ *Mfn1* ↑ *Nrf-2* ↑ *Mfn2* ↑ *Keap-1* ↓ *HO-1* ↑	Reduces cell apoptosis and modulates mitochondrial dysfunction.	

### 5.2. Plant Extracts

Diosgenin (DIO), a steroidal sapogenin found in plants such as Chinese yam, and fenugreek, exhibits antioxidant, anti-inflammatory, and anti-diabetic properties [104]. In diabetic models, DIO administration lowers fasting blood glucose levels and reduces serum creatinine and blood urea nitrogen levels. Histopathologically, DIO ameliorates renal structural damage by reducing tubular vacuolization, suppressing mesangial matrix expansion, and diminishing basement membrane thickening. Mechanistic study indicates that DIO activates CaMKK2, which in turn regulates the AMPK-mTOR axis to enhance autophagy and promotes via the *PINK1*-*Parkin* pathway, thereby restoring mitochondrial homeostasis. Notably, both pharmacological and genetic inhibition of CaMKK2 abolished these protective effects, confirming its essential role in mediating mitochondrial dynamics homeostasis.

Resveratrol, a naturally occurring polyphenol abundant in grapes, peanuts, berries, and other plant foods [105], has demonstrated protective potential against DKD. Research reveals that its mechanism involves the inhibition of *Drp1*-mediated mitochondrial fission through modulation of the PDE4D/PKA pathway. Under hyperglycemic conditions, phosphodiesterase 4D (PDE4D) activity is elevated, leading to suppression of the cAMP/PKA signaling cascade. This results in decreased phosphorylation of *Drp1* at Ser637 and enhanced mitochondrial fission activity. Resveratrol intervention effectively suppresses PDE4D, thereby restoring cAMP/PKA pathway function and increasing inhibitory phosphorylation of *Drp1*. This regulatory action attenuates mitochondrial fragmentation, ameliorates renal fibrosis and structural damage, and contributes to the improvement in DKD [82].

Germacrone, a sesquiterpenoid derived from turmeric, demonstrates renoprotective effects in DKD through multi-target mechanisms [106,107]. This agent exerts its effect by inhibiting ferroptosis through reductions in accumulation of lipid peroxides and free iron, attenuating mitochondrial ROS production, and reactivating mitophagy to clear damaged organelles, which in turn restores mitochondrial homeostasis, ATP production, and mitochondrial membrane potential [108]. Through these coordinated mechanisms, germacrone attenuates podocyte apoptosis and tubular injury, ultimately mitigating DKD progression.

Astragaloside IV (AS-IV), a key saponin from Astragalus membranaceus, exhibits multiple pharmacological activities, including anti-inflammatory, antioxidant, anti-apoptotic, and anti-fibrotic effects. In db/db mice with DKD, AS-IV treatment improves functional markers (reducing albuminuria and urinary N-acetyl-β-D-glucosaminidase) and alleviates histopathological injuries such as mesangial matrix expansion and tubular hypertrophy. Mechanistically, AS-IV inhibits diabetes- induced upregulation of mitochondrial fission proteins (*Drp1*, Fis1, MFF), thereby restoring mitochondrial dynamics by inhibiting excessive *Drp1*-mediated fission. These findings elucidate a mitochondrial-based mechanism for its renoprotective effects and support its potential translational relevance [109].

**Table 3:** Plant Extracts Targeting Mitochondrial Dynamic Homeostasis in Diabetic Kidney Disease.

Drug-	Model	Mechanism	Influence	References
DIO	HFD+STZ-induced male *SD* rats	*PINK1* ↑ *Parkin* ↑ *Opa1* ↑ *LC3II* ↑ *p62* ↓ *AMPK* ↑ *p-AMPK* ↑ *p-mTOR* ↓ *P70S6K* ↓ *p-P70S6K* ↓ *Drp1* ↓ *Mfn1/2* ↑	Lowers blood glucose, improves mitochondrial dynamics, enhances mitophagy.	[104]
	HG-induced HK-2 cells	*PINK1* ↑ *Parkin* ↑ *Opa1* ↑ *LC3II* ↑ *p62* ↓ *p-mTOR* ↓ *P70S6K* ↓ *p-P70S6K* ↓ *Drp1* ↓ *Mfn1/2* ↑	Reduces cellular apoptosis, improves mitochondrial dynamics, enhances mitophagy.	
Resveratrol	*db*/*db* male mice	*Drp1* ↓ *ROS* ↓ *PDE4D* ↓	Lowers blood glucose, alleviates fibrosis, suppresses oxidative stress, and mitigates mitochondrial dysfunction	[105]
	HG-induced GMCs cells	*Drp1* ↓ *ROS* ↓ *PDE4D* ↓	Reduces cell apoptosis, suppresses mitochondrial fission.	
Germacrone	*db*/*db* male mice	*Kim1* ↓ *Ngal* ↓ *PAI-1* ↓ *B2M* ↓ *COX-2* ↓ *ACSL4* ↓ *NOX1* ↓ *GPX4* ↑ *FTH1* ↑	Reducing iron death in the kidneys and restoring mitophagy	[108]
	HG-induced HK-2 cells	*ROS* ↓ *PINK1* ↑ *parkin* ↑ *Lc3II* ↑ *COX IV* ↓ *p62* ↓ *FTH1* ↓ *TOM20* ↓ *Tim23* ↓ *p-STING* ↓ *STING* ↓	Inhibiting ferroptosis, reducing cell apoptosis	
AS-IV	*db*/*db* male mice	*Drp1* ↓, *Fis-1* ↑ *MFF* ↑ *P* *ink1* ↑ *Parkin* ↑ *LC3II* ↑	Reduces urinary protein levels, alleviates renal injury, and restores the mitochondrial quality control network	[109]

### 5.3. Chinese Medicine Formulae

The Jin ChanYi ShenTong Luo Formula (LCYSTL), a multi-herbal traditional Chinese medicine composition comprising eight botanical components, exhibits significant renoprotective effects in a type 1 diabetic rat model [52]. JCYSTL-treated animals show improved renal function, including reduced serum creatinine, blood urea nitrogen uric acid, and albuminuria. JCYSTL attenuated renal injury, manifested as diminished renal interstitial fibrosis, suppressed inflammatory infiltration, and ameliorated tubular dilation. Mechanistically, JCYSTL exerts its benefits by modulating the hypoxia-inducible factor-1α (HIF-1α) signaling pathway and enhancing mitophagy. Under hyperglycemic conditions, JCYSTL activates HIF-1α signaling, which in turn promotes mitophagic clearance of damaged mitochondria via mitophagy, improves mitochondrial recovery, and suppresses apoptosis in tubular epithelial cells. These findings suggest that JCYSTL ameliorates DKD progression through coordinated activation of the HIF-1α/mitophagy axis, highlighting its potential as a multi-target therapeutic strategy.

The Yiquyangyin Huazhuo Tongluo (YYHT) formula, a traditional Chinese medicine, protects against podocyte injury in high glucose-induced DKD models by modulating mitophagy. In vitro, serum containing YYHT formula enhances podocyte mitophagy and upregulates podocyte-specific proteins Nephrin and Podocin. This efficacy correlates with improved mitophagic function. Mechanistic research reveals that high glucose induces podocyte injury via miR-21a-5p/*FoxO1*/*PINK1* axis: overexpression of miR-21a-5p in podocytes suppresses FoxO1 activity, downregulating *PINK1*/*Parkin* and impairing mitophagy. The YYHT formula counteracts this by inhibiting miR-21a-5p, thereby restoring FoxO1-driven *PINK1*/*Parkin* expression, reactivating mitophagy, clearing damaged mitochondria, and alleviating podocyte injury in DKD [110].

Compound XueShuanTong (CXST), a traditional Chinese medicine formulation comprising Panax notoginseng, Astragalus membranaceus, Salvia miltiorrhiza, and Scrophularia ningpoensis, has demonstrated significant protective effects in DKD models. CXST treatment significantly reduces proteinuria and improves renal function, while histopathology confirms attenuation of glomerular mesangial matrix expansion and podocyte injury. Mechanistically, CXST promotes podocyte mitophagy by activating the AMPK pathway and inhibiting mTOR signaling. As a central cellular energy sensor, AMPK activation inhibits mTOR, a key negative regulator of autophagy, thereby enhancing the clearance of damaged mitochondria under diabetic conditions. CXST reduces mitochondrial ROS overproduction and alleviates hyperglycemia-induced podocyte damage and apoptosis. These findings indicate that CXST ameliorates DKD progression through restoring mitochondrial homeostasis via AMPK/mTOR-mediated mitophagy [111].

The Modified Fenugreek Pill, a classic Chinese herbal formulation for diabetes, demonstrates protective effects against DKD by maintaining podocyte integrity. In diabetic rats, it reduces proteinuria, improves renal function, and attenuates pathological changes including glomerular basement membrane thickening and podocyte detachment. Mechanistic research shows that this formula activates pyruvate kinase M2 (PKM2), a key glycolytic enzyme, which in turn restores mitochondrial dynamic homeostasis in podocytes by inhibiting Drp1-mediated mitochondrial fission and enhancing Mfn2-mediated fusion. Consequently, it ameliorates hyperglycemia-induced mitochondrial fragmentation, preserves mitochondrial network integrity, and ultimately reduces podocyte apoptosis, and delays the DKD progression. These findings establish a novel link between energy metabolism and organelle homeostasis in diabetic kidney injury [112].

**Table 4:** Traditional Chinese Medicine Formulae Targeting Mitochondrial Dynamic Homeostasis in Diabetic Kidney Disease.

Drug	Model	Mechanism	Influence	References
JinChan YiShen TongLuo	Unilateral nephrectomy + STZ-induced male *SD* rats	The activity of mitochondrial respiratory chain complexes I, III, and IV ↑ *Bax* ↓ *C-Caspase3* ↓	Reduce urinary albumin, alleviate renal tubulointerstitial lesions, and improve mitophagy dysfunction	[52]
Yiquyangyin Huazhuo Tongluo	HG-induced MPC5 cells	*Nephrin* ↑ *Podocin* ↑ *FoxO1* ↑ *PINK1* ↑ *Parkin* ↑	Alleviates podocyte injury, promotes mitophagy.	[110]
Compound XueShuanTong	STZ-induced male *SD* rats	*Nephrin* ↑ *P62* ↓ *Podocin* ↑ *Beclin1* ↑ *LC3-II* ↑ *PINK1* ↑ *Parkin* ↑ *p-AMPK* ↑ *p-mTOR* ↓	Alleviates renal injury, ameliorates lipid metabolic dysfunction, promotes mitophagy.	[111]
	HG-induced MPC5 cells	*Nephrin* ↑ *P62* ↓ *Podocin* ↑ *Beclin1* ↑ *LC3-II* ↑ *PINK1* ↑ *Parkin* ↑ *p-AMPK* ↑ *p-mTOR* ↓	Alleviates podocyte injury, promotes mitophagy	
Modified Fenugreek Pill	*db*/*db* male mice	*CD2AP* ↑ *Nephrin* ↑ *Podocin* ↑ *P-cadherin* ↑ *MFF* ↓ *Mid51* ↓ *Fis1* ↓ *Mfn1/2* ↑ *PKM2* ↑ *PGC-1α* ↑ *Opa1* ↑	Reduces urinary protein levels, alleviates renal injury, and maintains mitochondrial homeostasis.	[112]
	AGEs-induced MPC5 cells	*ROS* ↓ *PKM2* ↑ *PGC-1α* ↑ *Opa1* ↑ *MFF* ↓ *Fis1* ↓ *Mfn1* ↑	Reduces cellular apoptosis, restores mitochondrial homeostasis.	

### 5.4. Mitochondrial-Target Agents

Elamipretide (MTP-131, SS-31) is a cationic tetrapeptide that selectively targets mitochondrial cardiolipin. It readily penetrates cell membranes, localizes to mitochondria, and exerts biological effects including mitochondrial structure stabilization, functional improvement, and redox regulation. Experimental studies in db/db mice, a model of type 2 DKD, show that Elamipretide treatment effectively delays disease progression and significantly improves renal function, as reflected by reduced urinary albumin excretion, lower serum creatinine and blood urea nitrogen, and ameliorated glucose control and insulin resistance. Histopathological analyses further confirm renal protection: periodic acid–Schiff (PAS) staining indicates suppressed glomerular mesangial matrix expansion. Mechanistically, Elamipretide binds to cardiolipin, normalizes pathologically elevated mitochondrial superoxide (mtROS) levels in diabetic kidneys, and subsequently mitigates oxidative stress-induced damage. It also stabilizes mitochondrial membrane integrity, rebalances mitochondrial dynamics (inhibiting fission and promoting fusion), enhances respiratory capacity and ATP production, and thereby suppresses tubular apoptosis and fibrosis-related signaling pathways such as TGF-β1/Smad3 pathway. Together, these findings underscore that precise modulation of mitochondrial superoxide by Elamipretide is central to its renoprotective action, providing strong experimental support for mitochondrial-targeted therapy in DKD [113].

MitoQ is a mitochondria-targeted antioxidant consisting of a ubiquinone moiety linked to a triphenylphosphonium (TPP) cation, which enables efficient accumulation within mitochondria and selective scavenging of mitochondrial-derived reactive oxygen species (ROS). In experimental models of DKD, MitoQ administration effectively improved renal function, as evidenced by reduced urinary albumin excretion and decreased expression of tubular injury markers such as β-NAG. Histopathological evaluation showed that MitoQ treatment alleviated renal structural damage: periodic acid–Schiff (PAS) staining indicated attenuated glomerular hypertrophy and mesangial matrix expansion, while transmission electron microscopy revealed preserved mitochondrial morphology—including reduced fragmentation—in tubular epithelial cells. Mechanistically, MitoQ was shown to activate the *Nrf2*/*PINK1* pathway, thereby enhancing mitochondrial quality control. This is achieved by promoting the nuclear translocation and transcriptional activity of Nrf2, leading to upregulation of *PINK1* expression, which in turn stimulates *PINK1*-dependent mitophagy and facilitates the clearance of damaged mitochondria. Inhibition of *Nrf2* (using pharmacological inhibitors) or knockdown of *PINK1* (with siRNA) abolished both the mitophagic response and the tubular protective effects of MitoQ, confirming that the Nrf2/*PINK1* axis is essential for its renoprotective action in DKD [114].

**Table 5:** Mitochondrial-Targeted Agents for Diabetic Kidney Disease.

Drug-	Model	Mechanism	Influence	References
Elamipretide	*db*/*db* male mice	*Mfn1* ↑ *LCLAT1* ↑ *Pla2* ↓	Stabilizes cardiolipin, reduces mitochondrial superoxide, and improves mitochondrial dynamic homeostasis.	[113]
MitoQ	*db*/*db* male mice	*Mfn1* ↑ *Drp-1* ↓ *P62* ↓ *LC3-II* ↑	Ameliorates renal injury and restores mitochondrial quality control.	[114]
	HG-induced HK-2 cells	*PINK1* ↑ *Parkin* ↑ *Nrf2* ↑ *Keap1* ↓ *Mfn1* ↑ *Drp1* ↓	Reduces mitochondrial fragmentation, decreases cellular apoptosis and ROS, and restores mitochondrial function.	

## 6. Summary and Outlook

Diabetic kidney disease (DKD), a prevalent and serious complication in patients with diabetes, involves multiple cellular and molecular mechanisms in its onset and progression. Growing evidence suggests that mitochondria and their dynamic regulation play a critical role in the pathogenesis of DKD, affecting cellular metabolism and stress responses. Given this central role, this review systematically and innovatively integrates characteristic changes in mitochondrial dynamics across different stages of DKD and its major constituent cell types. It focuses on the differential regulatory mechanisms of mitochondrial fusion, fission, and autophagy in podocytes, tubular epithelial cells, and other renal cell types. Furthermore, it comprehensively summarizes recent advances in the renoprotective effects of various traditional Chinese medicine formulas via modulation of mitochondrial dynamics. This work aims to provide novel theoretical insights and potential therapeutic targets for the precision treatment of DKD.

Despite this progress, several challenges and unanswered questions remain. First, the precise relationship between abnormalities in mitochondrial dynamics and specific stages of DKD requires further elucidation through well-designed clinical studies and mechanistic experiments. Second, the long-term efficacy and safety of interventions targeting mitochondrial dynamics need rigorous evaluation in clinical trials. Third, achieving cell-type specificity and minimizing off-target effects will be crucial for translating these strategies into clinically viable therapies.

Future research should focus on deepening our understanding of the molecular mechanisms underlying mitochondrial dynamics in different renal cell types and their crosstalk with other pathological processes in DKD. The integration of multi-omics approaches, including transcriptomics, proteomics, and metabolomics, with single-cell technologies will help unravel the complex regulatory networks governing mitochondrial homeostasis in the diabetic kidney. Furthermore, the development of personalized treatment strategies based on individual variations in mitochondrial function may represent a promising direction for precision medicine in DKD. In conclusion, while targeting mitochondrial dynamics opens new avenues for DKD diagnosis and therapy, its full clinical translation will require continued interdisciplinary efforts to bridge the gap between basic research and clinical application.

## Figures and Tables

**Table 1:** Summary of cellular pathogenesis in diabetic kidney disease.

Cell Type	Primary Trigger(s)	Core Pathological Changes	Key Molecular & Cellular Mechanisms	Functional & Structural Outcomes
Endothelial Cells	Hyperglycemia, AGEs, hemodynamic stress	Endothelial dysfunction, glycocalyx shedding, increased permeability, pro-inflammatory activation	NO ↓, ET-1 ↑ • Mitochondrial ROS ↑ VCAM-1/ICAM-1 ↑ • Dysregulated VEGF signaling	Microvascular leakage, disruption of endothelial podocyte crosstalk, initiation of glomerular inflammatory milieu
Podocytes	Albumin overload (due to endothelial leakage), direct glucotoxicity, metabolic stress	Foot process effacement, cytoskeletal rearrangement, apoptosis/detachment, impaired autophagy/mitophagy	Nephrin, podocin ↓ Drp1-mediated fission ↑ & ROS ↑ Aberrant mTOR signaling TGF-β/Smad3 activation	Proteinuria (loss of filtration barrier), podocyte depletion, denudation of glomerular basement membrane
Mesangial Cells	Injury signals from endothelium or podocytes (cytokines, ROS), metabolic substrate accumulation	Phenotypic activation (proliferation & hypertrophy), excessive ECM deposition, reduced contractility	PDGF, TGF-β1 signaling ↑ ROS/NLRP3 inflammasome activation Imbalanced mitochondrial dynamics (fission ↑, fusion ↓) MMPs/TIMPs imbalance	Mesangial matrix expansion, capillary lumen compression, progressive glomerulosclerosis
Tubular Epithelial Cells	Albumin overload, accumulated glycolytic metabolites, local ischemia/hypoxia	Brush border loss, vacuolization, epithelial-mesenchymal transition (EMT), apoptosis/necrosis	Enhanced fission (Drp1 activation) & suppressed fusion (*Opa1*/Mfn2 ↓) Impaired *PINK1*/*Parkin*-mediated mitophagy Activation of TGF-β/Smad, Wnt/β-catenin pathways	Tubular reabsorption or secretion dysfunction, tubular atrophy, interstitial inflammatory infiltration, interstitial fibrosis

## Data Availability

No new data were created or analyzed in this study. Data sharing is not applicable.
